# A possible variant of Bouveret's syndrome presenting as a duodenal stump obstruction by a gallstone after Roux-en-Y gastrectomy: a case report

**DOI:** 10.1186/1752-1947-3-7301

**Published:** 2009-05-28

**Authors:** Shruti Mittal, Robert P Sutcliffe, Ashish Rohatgi, Simon W Atkinson

**Affiliations:** 1 Department of Surgery, St Thomas' Hospital, Lambeth Palace Road, London SE1 7EH, UK

## Abstract

**Introduction:**

Bouveret's syndrome is characterized by gastric outlet obstruction due to a gallstone in the duodenum, usually in association with a cholecystoduodenal fistula.

**Case presentation:**

We report the case of a 69-year-old Caucasian man who developed duodenal stump obstruction due to an impacted gallstone after having previously undergone Roux-en-Y gastrectomy.

**Conclusions:**

Duodenal stump obstruction after Roux-en-Y gastrectomy is rare, and may be difficult to manage. Patients who present with upper gastrointestinal or pancreatobiliary pathology after previous gastric surgery should be managed in centres with the availability of appropriate endoscopic and surgical experience.

## Introduction

Bouveret's syndrome is a rare complication of gallstone disease that is characterized by gastric outlet obstruction secondary to an impacted gallstone in the duodenum [1]. This is usually in association with a cholecystoduodenal fistula [1]. We present an unusual case of duodenal stump obstruction by a gallstone in a patient who had previously undergone Billroth II gastrectomy with Roux-en-Y reconstruction.

## Case presentation

**Figure 1:**
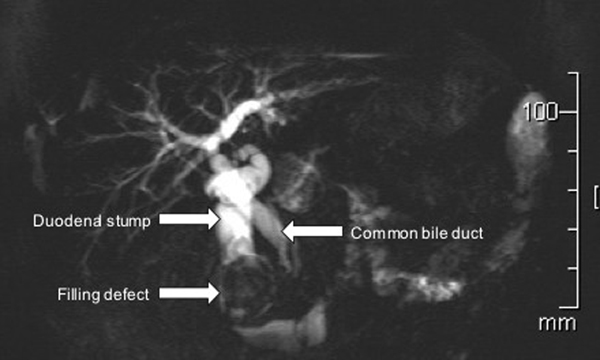
**Magnetic resonance cholangiopancreatography (a). Images showing a large filling defect in the duodenal stump, which had caused duodenal stump obstruction and extrinsic bile duct compression**.

A 69-year-old Caucasian man presented with intermittent upper abdominal pain, nausea and bloating for the previous 12 months. There was no history of fever, jaundice, pale stools or dark urine. His past medical history included a Billroth II gastrectomy and Roux-en-Y reconstruction for peptic ulcer disease 11 years earlier. On examination, he was haemodynamically stable and afebrile. Abdominal examination was unremarkable. Laboratory tests, including liver function tests and inflammatory markers were all normal. Transabdominal ultrasound examination revealed gallstones and intra- and extrahepatic biliary dilatation. Endoscopic retrograde cholangiopancreatography (ERCP) was unsuccessful. Magnetic resonance cholangiopancreatogram (MRCP) and computed tomography (CT) confirmed gallstones within the gallbladder and biliary dilatation to the level of the ampulla, and demonstrated a 4 × 3 cm low density filling defect in the second part of the duodenum (Figure 1).

**Figure 2:**
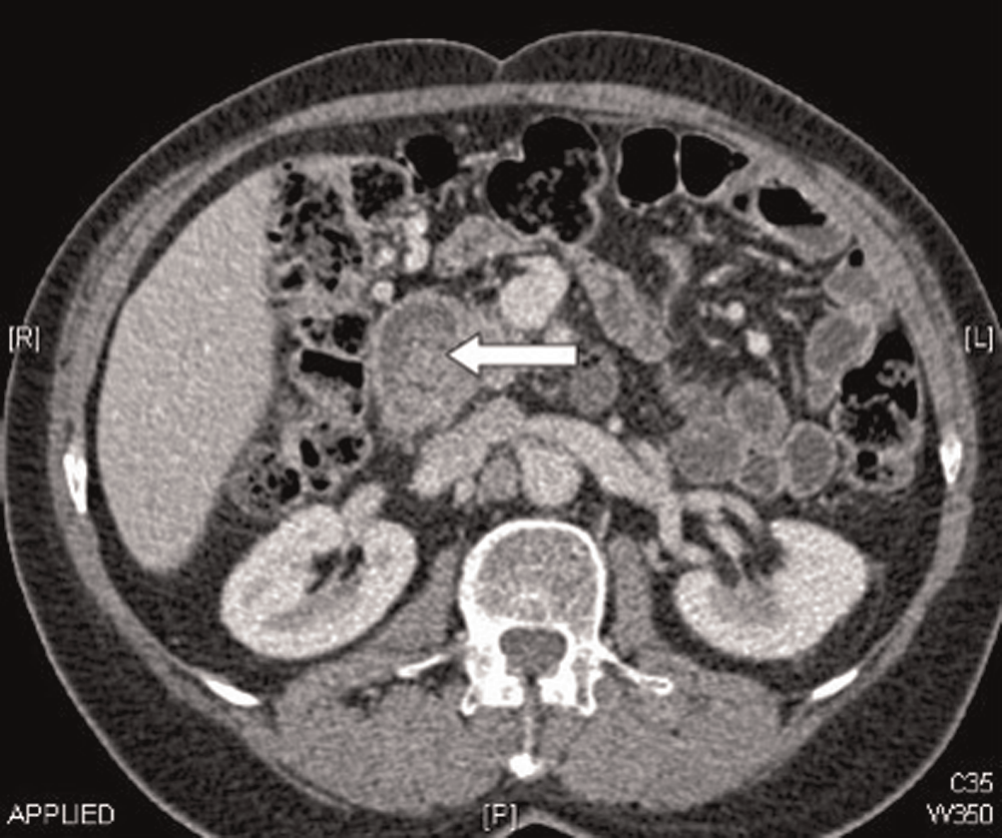
**Computed tomography (b). Images showing a large filling defect in the duodenal stump, which had caused duodenal stump obstruction and extrinsic bile duct compression**.

After he was admitted to a specialist centre, endoscopic ultrasonography and repeat ERCP were attempted but failed due to inability to cannulate the duodenal limb of the Roux-en-Y. Two hours after the procedure he developed severe, generalized abdominal pain and hypotension. This raised the possibility of iatrogenic intestinal perforation. However, it was decided to proceed directly to laparotomy rather than repeat imaging, since it was apparent that surgical exploration would be required in the event of unsuccessful ERCP. During the operation, there was no evidence of intestinal perforation. A 3.5 cm gallstone was found proximal to a stricture in the second part of the duodenum. There was no evidence of a cholecystoduodenal fistula. The duodenal papilla and pancreas were normal. The common bile duct, which was dilated due to extrinsic compression by the intraduodenal gallstone, was explored and found to be normal, and contained no stones. The gallstone in the duodenum was extracted via a duodenotomy, the duodenal stricture was dilated digitally and a cholecystectomy was performed. A t-tube was placed in the common bile duct. He made an uneventful postoperative recovery and was discharged 10 days later after a normal t-tube cholangiogram. The t-tube was removed six weeks post-operatively.

## Discussion

The diagnosis and management of suspected biliary pathology in patients following a Billroth II Roux-en-Y gastrectomy is a considerable challenge. Non-invasive investigations, such as CT or MRCP may be helpful to detect intraductal stones and exclude other pathologies. In view of the distorted anatomy, an ERCP should be performed by an experienced endoscopist in a centre with access to surgical and radiological support [2]. Retrograde cannulation of the papilla may be possible in some patients, but in cases of difficulty, the procedure should be abandoned early to reduce the risk of small bowel perforation. A forward-viewing endoscope may be safer than a side-viewing endoscope in this group of patients and has been recommended previously [3]. Percutaneous transhepatic cholangiography followed by sequential dilatation and percutaneous cholangioscopy has been reported to be successful, and may be an option for patients unfit for surgery [4]. Surgical exploration of the common bile duct is necessary where endoscopic and percutaneous techniques are unsuccessful. Reoperation in these patients poses a significant risk of inadvertent gastrointestinal injury due to the presence of adhesions.

Bouveret's syndrome is a rare complication of gallstone disease [1], and is characterized by passage of a large stone through a cholecystoduodenal fistula into the duodenum, which becomes impacted. Patients typically present with features of gastric outlet obstruction. A barium meal and/or endoscopy may confirm the diagnosis, and endoscopic disimpaction may be possible, but surgery is frequently necessary. Cholecystectomy carries no benefit in these cases, and is often very difficult and should be avoided.

This case is an unusual variant of Bouveret's syndrome with some interesting differences. There have been reports of gallstones causing afferent loop obstruction after Billroth II gastrectomy in patients with a cholecystoduodenal fistula [5]. In our patient, a large gallstone became impacted in the duodenum in the absence of a fistula. The most likely explanation in our patient is that multiple small stones passed from the gallbladder into the duodenum via the papilla and became enlarged by a process of accretion as a result of the stricture in D2, ultimately leading to obstruction of the duodenal stump. The aetiology of the duodenal stricture was unclear, but it may have been inflammatory or due to adhesions. Since the patient had previously had a Roux-en-Y reconstruction, the gallstone caused duodenal stump obstruction, resulting in abdominal pain, rather than gastric outlet obstruction as originally described by Bouveret. There was no evidence of a cholecystoduodenal fistula, and therefore cholecystectomy could be safely undertaken in this patient.

## Conclusion

Investigation of the biliary tree after Roux-en-Y gastrectomy presents a significant challenge. Noninvasive imaging, such as CT or MRCP may provide useful information to guide management. ERCP may be possible but should be performed by an experienced endoscopist in a specialist centre with availability of radiological and surgical expertise. In the presented case, an impacted gallstone in the duodenal stump could not be removed endoscopically and required surgical intervention.

## Consent

Written informed consent was obtained from the patient for publication of this case report and any accompanying images. A copy of the written consent is available for review by the Editor-in-Chief of this journal.

## Competing interests

The authors declare that they have no competing interests.

## Authors' contributions

SM and RS collected the data and drafted the manuscript. AR performed a literature review and helped draft the manuscript. SA conceived the study. All authors have read and approved the final manuscript.
